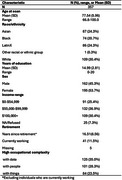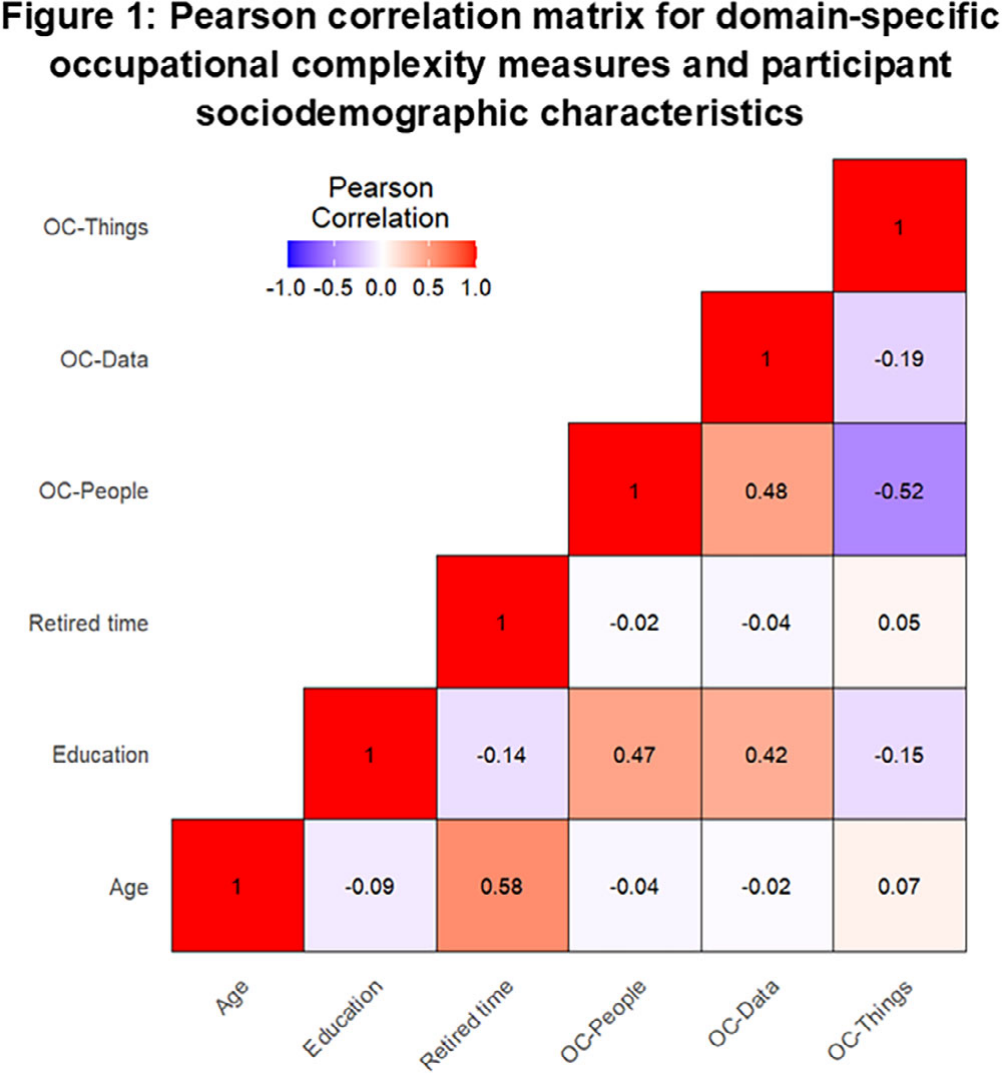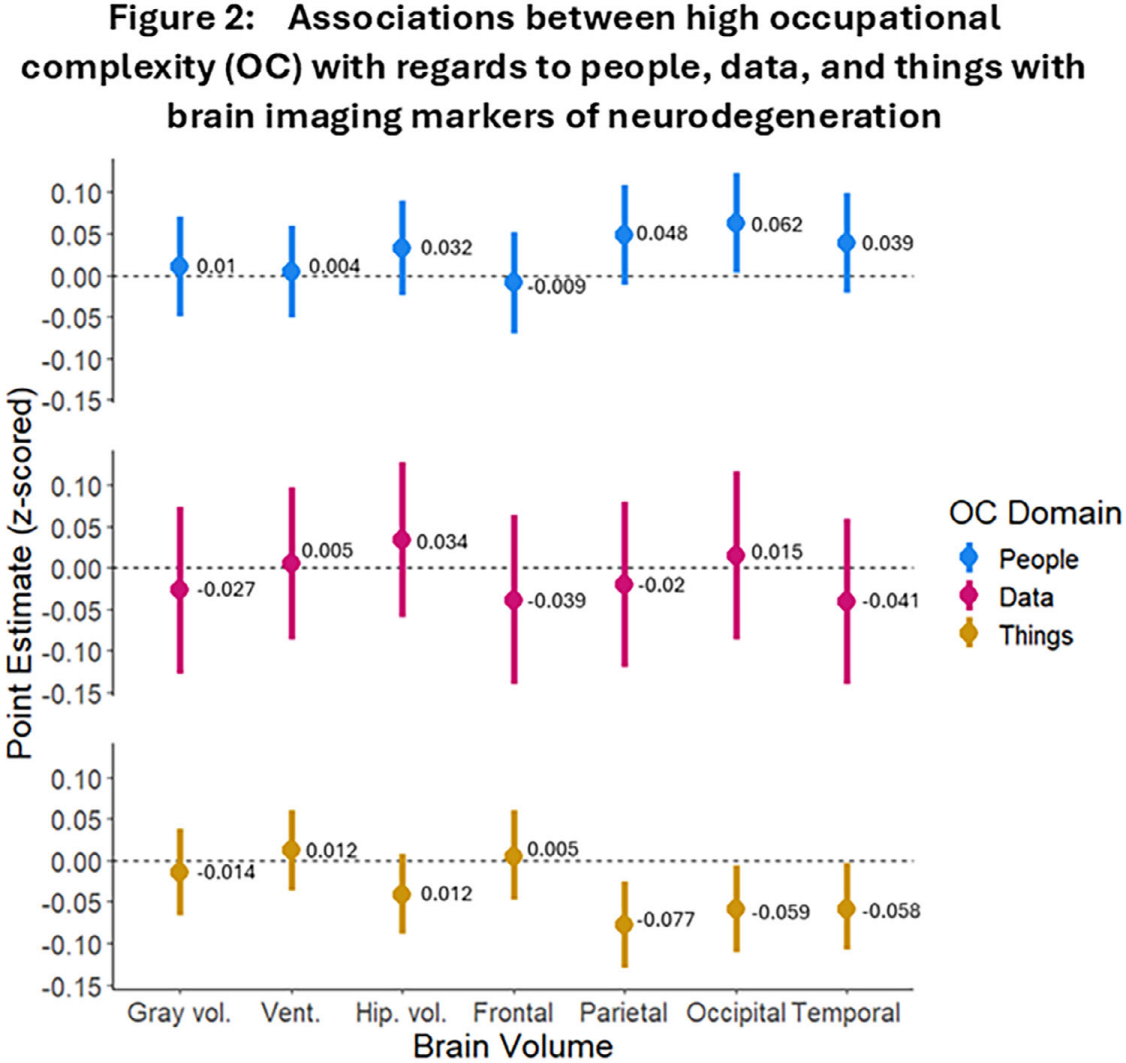# Occupational complexity of work and brain volume in an older multi‐ethnic sample

**DOI:** 10.1002/alz70860_107427

**Published:** 2025-12-23

**Authors:** Molly R. LaPoint, L. Paloma Rojas‐Saunero, Hilary L. Colbeth, Chloe W. Eng, Joseph N. Roscoe, Rachel A. Whitmer, Elizabeth Rose Mayeda, M. Maria Glymour, Paola Gilsanz

**Affiliations:** ^1^ Kaiser Permanente Northern California Division of Research, Pleasanton, CA, USA; ^2^ UCLA Fielding School of Public Health, University of California, Los Angeles, CA, USA; ^3^ University of California, Davis, Davis, CA, USA; ^4^ Stanford University, Stanford, CA, USA; ^5^ Boston University School of Public Health, Boston, MA, USA

## Abstract

**Background:**

Occupational complexity (OC) is associated with cognitive health among older adults, but the association between OC domains (e.g. people, data, and things) and brain health is unclear.

**Method:**

We analyzed data from a subset of Kaiser Healthy Aging and Diverse Life Experiences (KHANDLE) study participants (*N* = 357) who were randomly sampled to complete a 3T MRI. Self‐reported main lifetime occupation was linked to OC scores in domains of people, data, and things obtained from the 1970 US Census Dictionary of Occupational Titles. Measures of cortical gray matter, hippocampus, ventricular, frontal, occipital, parietal, and temporal lobe volumes were residualized on intracranial volume and z‐scored. Pearson correlations investigated relationships between OC domains and continuous covariates. Separate linear regression models estimated the association between domain‐specific OC and imaging markers adjusting for current income range, race/ethnicity, sex, years of education, length of time since retirement (zero for those still working), and age at scan using robust standard errors.

**Result:**

Mean age was 77.54 (Table 1). Higher OC‐things was moderately negatively correlated with OC‐people (*r* = ‐0.52, *p* <0.01) and weakly negatively correlated with OC‐data (*r* = ‐0.19, *p* <0.01; Figure 1).

Higher OC‐people was positively associated with occipital lobe volume (β=0.062, 95%CI: 0.002, 0.123). Point estimates for hippocampal volume (β=0.032, 95%CI: ‐0.024, 0.089), parietal (β=0.048, 95%CI: ‐0.012, 0.123), and temporal (β=0.048, 95%CI: ‐0.021, 0.098) lobe volume were positive but not significant (Figure 2). There were no associations between OC‐people and other brain regions nor between higher OC‐data and any brain region volume. Higher OC‐things was inversely associated with parietal (β=‐0.077, 95%CI: ‐0.129, ‐0.025), occipital (β=‐0.059, 95%CI: ‐0.111, ‐0.006), and temporal (β=‐0.058, 95%CI: ‐0.107, ‐0.004) lobe volumes. Higher OC‐things was also inversely associated with hippocampal volume (β=‐0.041, 95%CI: ‐0.089 0.008) but not significantly so.

**Conclusion:**

Higher complexity of work with people was inversely correlated with complexity of work with things. Higher complexity of work with things was associated with smaller parietal, occipital, and temporal lobe volumes. Further research in larger datasets with repeated measures are needed to better understand the how occupational complexity is associated with brain health across the lifecourse.